# The specific ex vivo released cytokine profile is associated with ischemic stroke outcome and improves its prediction

**DOI:** 10.1186/s12974-019-1691-1

**Published:** 2020-01-06

**Authors:** Elzbieta Klimiec-Moskal, Marcin Piechota, Joanna Pera, Kazimierz Weglarczyk, Agnieszka Slowik, Maciej Siedlar, Tomasz Dziedzic

**Affiliations:** 1 Department of Neurology, Jagiellonian University Medical College, ul. Botaniczna 3, 31-503 Kraków, Poland; 2 Department of Molecular Neuropharmacology, Maj Institute of Pharmacology, Polish Academy of Sciences, ul. Smetna 12, 31-343 Krakow, Poland; 3 Department of Clinical Immunology, Institute of Paediatrics, Jagiellonian University Medical College, ul. Wielicka 265, 30-663 Krakow, Poland

**Keywords:** Stroke, Cytokine, Outcome, Inflammation, Biomarker, Prediction

## Abstract

**Background:**

Inflammation is associated with poor outcome after stroke. A relationship between ex vivo cytokine synthesis and stroke outcome remains unclear. We explored an association between ex vivo cytokine release, circulating interleukin (IL)-6 as a marker of systemic inflammation, and stroke prognosis. We assessed the utility of ex vivo synthesized cytokines for predicting stroke outcome.

**Methods:**

We collected blood from 248 ischemic stroke patients and stimulated it ex vivo with lipopolysaccharide. We measured concentration of synthesized cytokines (TNFα, IP-10, IL-1β, IL-6, IL-8, IL-10, and IL-12) and plasma IL-6. We assessed functional outcome 3 months after stroke using the modified Rankin Scale. To assess the prognostic ability of cytokines, we applied multivariate logistic regression, cluster analysis, and construction of multimarker score.

**Results:**

Decreased release of IP-10, TNFα, IL-1β, and IL-12; increased release of IL-10 and IL-8; and higher plasma IL-6 level were associated with poor outcome. Cluster analysis identified three groups of patients with distinct cytokine profiles. The group with the worst outcome demonstrated high synthesis of IL-10, IL-8, IL-1β, and IL-6 and low synthesis of IL-12, IP-10, and TNFα accompanied by high circulating IL-6 level. The group with the best prognosis showed high synthesis of TNFα, IP-10, IL-12, IL-1β, and IL-6; low synthesis of IL-10 and IL-8; and low plasma IL-6. Patients with intermediate outcome had low synthesis of all cytokines accompanied by low circulating IL-6. We constructed a multimarker score composed of ex vivo released IL-12, IL-10, TNFα, and plasma IL-6. Addition of this score to clinical variables led to significant increase in c-statistic (0.81 vs 0.73, *p* = 0.02) and net reclassification improvement.

**Conclusion:**

The decreased ex vivo release of pro-inflammatory cytokines and increased release of IL-10 and IL-8 are related to poor outcome after stroke. Cytokine-based multimarker score adds prognostic value to clinical model for predicting stroke outcome.

## Background

Acute ischemic stroke is accompanied by both immunodepression and systemic inflammation [1, 2]. Post-stroke immunodepression refers to suppression of both innate and adaptive immune response and is marked by transient lymphopenia, functional deactivation of monocytes, and splenic atrophy. At the cytokine level, immunodepression is reflected in humans by the reduced ex vivo tumor necrosis factor alpha (TNFα) synthesis after whole blood or monocyte stimulation with endotoxin [3, 4] whereas circulating interleukin-6 (IL-6) is the main cytokine related to systemic inflammation [5].

Numerous studies showed that elevated blood level of IL-6 is an independent predictor of poor functional outcome after stroke [6, 7]. However, the addition of IL-6 to a validated, clinical-based stroke prognostic model did not improve the prediction of poor outcome. Much less is known about the prognostic significance of ex vivo cytokine production in stroke patients. Few studies suggest that the reduced ex vivo TNFα and interferon-gamma-inducible protein 10 (IP-10) synthesis after endotoxin challenge is associated with poor functional outcome after stroke [8, 9].

A better understanding of interactions between cytokine synthesis and stroke prognosis is important for at least two reasons. First, cytokine synthesis could be a potential therapeutic target in acute stroke [10]. Second, individual cytokines released by blood cells or their combination might serve as a biomarker to stratify risk of unfavorable outcome after stroke.

In this study, we aimed to explore an association between ex vivo cytokine synthesis, circulating IL-6 as a marker of systemic inflammation and stroke prognosis. We also examined the individual and collective utility of ex vivo synthesized cytokines for predicting stroke outcome.

## Methods

### Participant and clinical assessment

We prospectively recruited participants from consecutive stroke patients admitted to the Department of Neurology, University Hospital in Krakow, Poland, between October 2016 and September 2018. The inclusion criteria were (1) ischemic stroke, (2) admission within 24 h from symptom onset, (3) pre-stroke modified Rankin Scale (mRS) score 0–2, (4) National Institute of Health Stroke Scale (NIHSS) score on admission >3, and (5) informed consent. We excluded patients with chronic inflammatory, autoimmune, or cancerous diseases. Bioethics Committee of Jagiellonian University approved the study protocol (decision number: 122.6120.249.2016), and each patient gave an informed consent.

For ischemic stroke diagnosis, we used the updated definition from the American Heart Association/American Stroke Association [11]. Each patient underwent the head CT scan on admission. To assess stroke severity on admission, we used NIHSS score [12]. We used the TOAST criteria to determine stroke etiology [13]. We evaluated functional outcome 3 months after stroke with the mRS [14]. We defined poor outcome as a mRS score 3–6.

### Laboratory assays

We collected venous blood in heparinised tubes (Sarstedt, Germany) at day 3 after stroke onset. To avoid diurnal variation in cytokine production, we took blood between 7.00 and 7.30 am. Subsequently, we diluted the whole blood by 1:5 in sterile RPMI 1640 medium supplemented with l-glutamine (Sigma Aldrich, St. Louis, MO, USA) and we stimulated it in sterile tubes (Lonza, Walkersville, MD, USA) with lipopolysaccharide (LPS 10 ng/mL, E. coli 0111:B4, Sigma Aldrich, St. Louis, MO, USA) at 37 °C and 5% CO2. Based on previous publications [9, 15], we stimulated blood for 4 h for TNFα and IP-10 and 24 h for interleukin IL-1β, IL-6, IL-8, IL-10, and IL-12p70. We removed supernatants and stored them at − 80 °C.

To measure concentrations of TNFα and IP-10, we used commercially available ELISA kits from R&D Systems (Minneapolis, MN, USA). We measured IL-1β, IL-6, IL-8, IL-10, and IL-12p70 concentration using a cytometric bead array immunoassay (Human Inflammatory Kit, BD Bioscience, San Diego, CA, USA). To measure the level of IL-6 in plasma, we used ELISA kits from R&D Systems (Minneapolis, MN, USA) with a detection limit of 0.11 pg/ml.

### Statistical analyses

To better understand an association between cytokines and the poor outcome, we aimed to implement different statistical approaches.

As a first step, we compared baseline characteristics and cytokine levels between the group with good outcome and the group with poor outcome using χ2 test for proportions and Mann–Whitney *U* test for continuous variables. To find independent predictors of outcome, we performed logistic regression analyses. We standardized cytokines to the same scale (mean = 0, SD = 1) to facilitate comparison between them. We used univariate analysis to determine clinical and laboratory predictors. Then, cytokines with *p* < 0.05 in the univariate regression were assessed individually in backward elimination models adjusted for significant clinical predictors. We used a retention value of *p* < 0.05. Finally, we performed a backward elimination model containing all cytokines significant in the univariate analysis adjusted for clinical predictors.

In the second step, we wanted to divide subjects into a small number of groups with different cytokine concentrations and test whether any specific profile could be related to the poor outcome. Before clustering, we log transform cytokines because they were right-skewed and then we standardize them to the same scale (mean = 0, SD = 1). To check collinearity between cytokines, we calculated Pearson’s correlation coefficient and Spearman’s rho using a threshold of 0.7 as an indicator of high collinearity [16]. We grouped patients using agglomerative hierarchical clustering with Ward’s method of minimum variance and the squared Euclidean distance metric [17]. We considered this method appropriate for our purposes as it could construct relatively compact clusters with emphasis on differences between observations [18]. Clustering was unsupervised and blinded to outcome. We determined the optimal number of clusters based on 30 indices provided by NbClust package in R [19]. To better characterize cytokine profiles, we compared clusters with Kruskal-Wallis test followed by *post-hoc* Dunn’s test. Next, to determine an association between clusters and the outcome, we performed uni- and multivariate logistic regressions. The latter was adjusted for previously identified clinical predictors.

In the final step, we constructed the multimarker score to further evaluate the prognostic value of investigated cytokines. We included cytokines that remained significant in the final backward elimination model. The score was defined as:

$$ \mathrm{Score}=\left({\beta}_{\mathrm{A}}\times \mathrm{cytokineA}\right)+\left({\beta}_{\mathrm{B}}\times \mathrm{cytokineB}\right)+\left({\beta}_{\mathrm{C}}\times \mathrm{cytokineC}\right),\mathrm{etc}., $$

where β_A_, β_B_, and β_C_ stand for regression β-coefficients for cytokines A, B, and C, respectively, from the backward regression model. We used a model with two most important predictors of outcome: stroke severity on admission and age as the best-fit clinical model [20]. Then, we compared the sole best-fit clinical model with the best-fit clinical model containing the multimarker score. We also compared the best-fit clinical model containing the multimarker score with best-fit clinical models containing individual cytokines. To evaluate the ability of models to discriminate between good and poor outcome, we calculated areas under receiver operator curves (AUC). An AUC of 1 indicates perfect discrimination and 0.5 no discrimination. We also assessed calibration with the Hosmer-Lemeshow χ2 statistic. Then, we calculated the integrated discrimination improvement (IDI), a measure of the model to improve average sensitivity without reducing average specificity [21]. To assess the ability of the score to reclassify risk, we calculated net reclassification improvement (NRI) metric [21]. Similarly to the previous study [22], we used clinically relevant thresholds (< 10%, 10–50%, 50–90%, > 90%) for the predicted probability of the poor outcome. The thresholds are based on the assumption that one would need to be very certain of a good or poor outcome before avoiding treatment such as thrombolysis or selecting patients for palliative care only. We also calculated a continuous NRI [23].

Statistical analyses were performed in STATA version 14 (StataCorp, College Station, TX) and packages “NbClust” [17] and “PredictABEL” [24] in R Statistical Software (Foundation for Statistical Computing, Vienna, Austria).

### Results

### Patients characteristic

We recruited 250 patients into the study. Information on 3-month functional status was available for 248 patients, and they were included into further analyses (median age 69 years, IQs 61–79; 41.1% female; median NIHSS score 9, IQs 5–17). From the final cohort, 111 participants (44.8%) had a poor 3-month functional outcome. Table 1 shows comparisons of baseline characteristics and cytokine levels between the group with good outcome and the group with poor outcome.

**Table 1:** Comparison of baseline characteristics and levels of cytokines between good and poor outcome groups

	Good outcome (mRS 0-2) *n* = 137	Poor outcome (mRS 3-6) *n* = 111	*p* value
Age, median (IQs)	66 (58–75)	72 (63–81)	<0.01
Female, *n* (%)	53 (38.7)	49 (44.1)	0.75
Hypertension, *n* (%)	104 (75.9)	90 (81.1)	0.33
Diabetes mellitus, *n* (%)	35 (25.6)	35 (31.5)	0.30
Atrial fibrillation, *n* (%)	40 (29.2)	31 (27.9)	0.83
Myocardial infarction, *n* (%)	17 (12.4)	17 (15.3)	0.51
Previous stroke, *n* (%)	14 (10.2)	16 (14.4)	0.31
Current smoking, *n* (%)	37 (27.0)	27 (24.3)	0.63
NIHSS score on admission, median (IQs)	6 (4–13)	15 (7–19)	<0.01
Thrombolysis, *n* (%)	77 (56.2)	61 (55.0)	0.84
Thrombectomy, *n* (%)	39 (28.5)	26 (23.4)	0.37
Etiology			0.17
Large vessel disease, *n* (%)	29 (21.2)	37 (33.3)	
Small vessel disease, *n* (%)	9 (6.6)	4 (3.6)	
Cardioembolic, *n* (%)	44 (32.1)	29 (26.1)	
Undetermined, *n* (%)	49 (35.8)	39 (35.1)	
Other, *n* (%)	6 (4.4)	2 (1.8)	
Cytokines			
Ex vivo stimulation			
IL-12p70, pg/ml, median (IQs)	5.6 (1.7–11.0)	3.0 (0.0–5.2)	<0.01
IL-10, pg/ml, median (IQs)	44.4 (32.0–69.3)	65.6 (40.4–89.4)	<0.01
IL-6, pg/ml, median (IQs)	11611 (8279–16298)	11534 (8013–17757)	0.96
IL-1β, pg/ml, median (IQs)	1611 (1101–2313)	1323 (797–2095)	0.01
IL-8, pg/ml, median (IQs)	1528 (953–2276)	2256 (1237–3459)	<0.01
TNFα, pg/ml, median (IQs)	2570 (1785–3793)	2071 (1518–2835)	<0.01
IP-10, pg/ml, median (IQs)	474 (25–832)	288 (138–534)	<0.01
Plasma			
IL-6, pg/ml, median (IQs)	3.2 (1.9–6.5)	8.9 (4.6–25.2)	<0.01

*IQs* interquartiles, *NIHSS* National Institutes of Health Stroke Scale

Patients with poor outcome were older and had higher NIHSS score on admission. They had the increased release of IL-10 and IL-8 and the decreased release of IL-12p70, IL-1β, TNFα, and IP-10 after ex vivo stimulation compared to the group with good outcome. Plasma level of IL-6 was also higher in the patients with worse outcome.

In the univariate analysis, older age and higher NIHSS score on admission were only clinical predictors of poor outcome (Table 2). Among cytokines, higher release of IL-10 and IL-8 after ex vivo stimulation as well as higher plasma level of IL-6 was associated with worse outcome. In contrast, the higher release of IL-12p70, IL-1β, TNFα, and IP-10 was related to the decreased risk of poor outcome. These results remained significant after adjustment for age and stroke severity. However, when we forced all cytokines into the backward elimination model adjusted for clinical predictors, only IL-12p70, IL-10, TNFα, and plasma IL-6 levels remained independent laboratory predictors of outcome.

**Table 2:** Predictors of poor outcome identified in the univariate analysis and results of backward elimination models

	Univariate analysis			Multivariate analysis of individual cytokines adjusted for age and stroke severity			Multivariate analysis of all cytokines adjusted for age and stroke severity		
	OR	95% CI	*p* value	OR	95% CI	*p* value	OR	95% CI	*p* value
Age	1.04	1.02–1.06	<0.01	1.04	1.02–1.06	<0.01	1.03	1.01–1.06	0.01
NIHSS on admission	1.12	1.07–1.16	<0.01	1.12	1.07–1.17	<0.01	1.09	1.04–1.14	<0.01
Cytokines*									
Ex vivo stimulation									
IL-12p70	0.46	0.31–0.67	<0.01	0.50	0.34–0.74	<0.01	0.55	0.36–0.84	<0.01
IL-10	1.55	1.17–2.05	<0.01	1.49	1.11–2.00	<0.01	1.83	1.32–2.56	<0.01
IL-1β	0.69	0.52–0.90	<0.01	0.74	0.55–0.99	0.04	0.85	0.60–1.20	0.36
IL-8	1.75	1.22–2.51	<0.01	1.54	1.06–2.22	0.02	1.28	0.80–2.05	0.31
TNFα	0.62	0.46–0.84	<0.01	0.67	0.49–0.92	0.01	0.64	0.44–0.92	0.02
IP-10	0.61	0.44–0.84	<0.01	0.65	0.47–0.90	0.01	0.89	0.59–1.34	0.58
Plasma									
IL-6	2.68	1.64–4.39	<0.01	1.86	1.17–2.94	< 0.01	1.59	1.02–2.47	0.04

*Cytokines were standardized to the same scale (mean = 0; SD = 1)

NIHSS – National Institutes of Health Stroke Scale; OR- odds ratio; 95% CI- 95% confidence interval

### Cluster analysis

We included plasma level of IL-6 and the following ex vivo produced cytokines: IL-12p70, IL-10, IL-6, IL-8, IL-1β, TNFα, and IP-10 into cluster analysis. Pearson’s correlation coefficients or Spearman’s rho between included cytokines did not exceed the threshold of 0.7.

We compared different numbers of clusters (maximal number of 6) based on 30 indices provided by NbClust package. The three-cluster solution showed the best performance (Additional file 1: Figure S1). Cluster characteristics are available in the Additional file 2: Table S1.

**Fig. 1:**
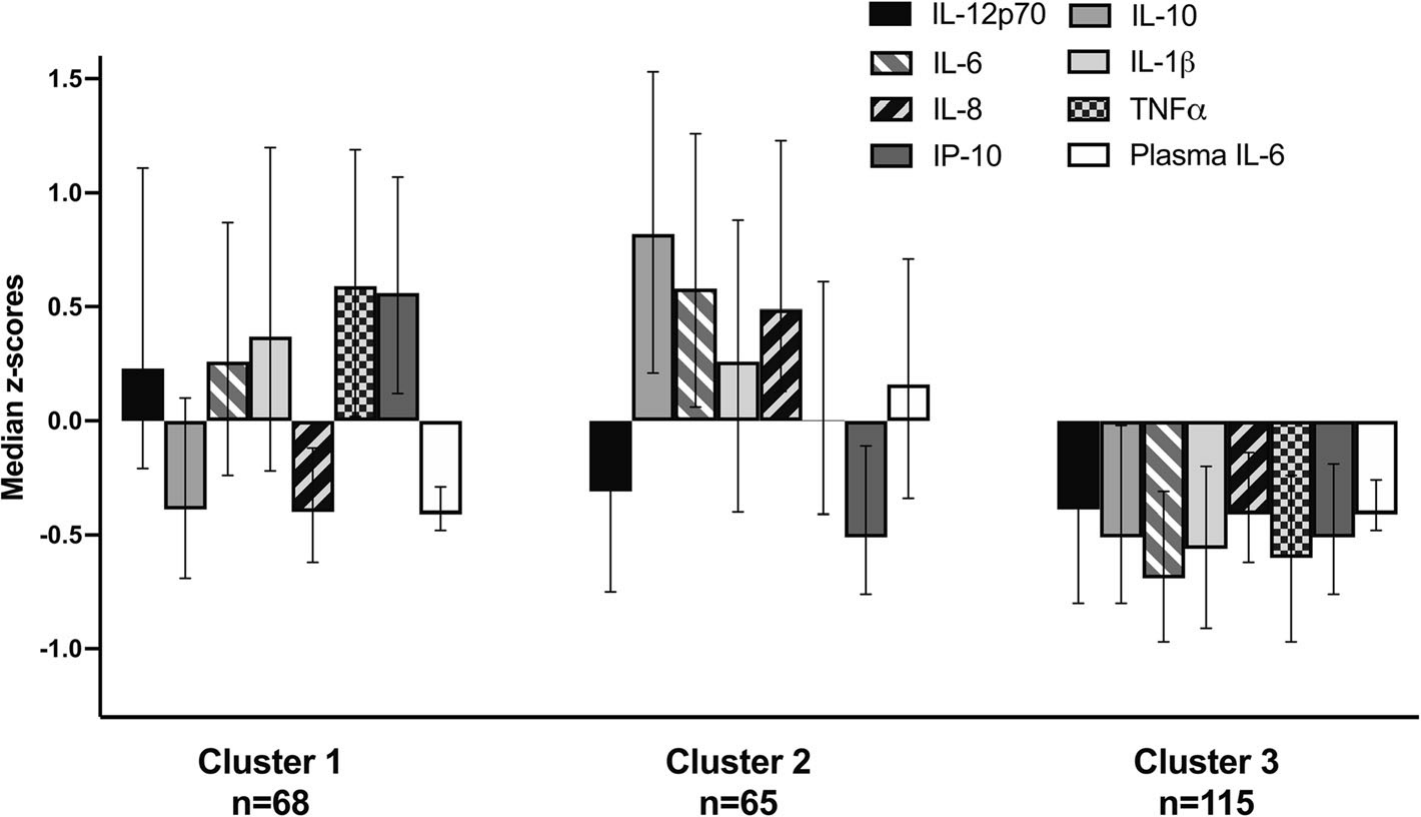
Results of cluster analysis. *Y* axis: median z-scores of eight clustered cytokines. Error bars represent interquartiles. *X* axis: clusters. IL—interleukin; IP-10—interferon-gamma-inducible protein 10; TNFα—tumor necrosis factor alpha

In the unsupervised, blinded to outcome analysis, we obtained three clusters with the different pattern of cytokine concentrations (Fig. 1). Cluster 1 (*n* = 68) had relatively high release of IL-12p70, TNFα, IP-10, IL-6, and IL-1β; low synthesis of IL-10 and IL-8; and low level of plasma IL-6. Cluster 2 (*n* = 65) demonstrated relatively high release of IL-10, IL-8, IL-1β, and IL-6; low release of IL-12p70, TNFα, and IP-10; and elevated level of plasma IL-6. Cluster 3 (*n* = 115) showed relatively low release of all cytokines after blood stimulation accompanied by the low level of plasma IL-6.

Kruskal-Wallis test followed by post-hoc characterization supported our visual observations (Additional file 2: Table S1). Cluster 1 had the highest release of IL-12p70 (*p* < 0.01), TNFα (*p* < 0.01), and IP-10 (*p* < 0.01) among all clusters. Cluster 2 had higher release of IL-10 (*p* < 0.01), IL-8 (*p* < 0.01), and plasma level of IL-6 (*p* < 0.01) compared to other clusters. Cluster 3 was characterized by the lowest release of IL-6 (*p* < 0.01), IL-1β (*p* < 0.01), and TNFα (*p* < 0.01) among clusters. There were no differences between cluster 1 and 3 in the plasma level of IL-6 (*p* = 1.00) and release of IL-10 (*p* = 0.37) and IL-8 (*p* = 0.98).

The 3-month functional outcome differed between three clusters. We observed the lowest rate of poor outcome in cluster 1, the highest in cluster 2, and intermediate in cluster 3 (25%, 69.2%, and 42.6%, respectively). When we used cluster 1 as a reference, the risk of poor outcome was increased 4.79-times (95%CI 2.13–10.78, *p* < 0.01) for cluster 2 and 2.14-times (95%CI 1.05–4.37, *p* = 0.04) for cluster 3 in the analysis adjusted for age and stroke severity.

### Multimarker score

To construct multimarker score, we used four cytokines that remained significantly associated with poor outcome in the final backward elimination model: IL-12p70, IL-10, TNFα, and plasma IL-6. Table 3 shows the β-coefficients of included cytokines and predictive values of multimarker score.

**Table 3:** Multimarker score and prediction of poor outcome

	Poor outcome	*p* value
Cytokines included in the score*		
IL-12p70, Coef (95%CI)	− 0.60 (− 1.03 to − 0.17)	<0.01
IL-10, Coef (95%CI)	0.61 (0.27–0.94)	<0.01
TNFα, Coef (95%CI)	− 0.45 (− 0.82 to − 0.08)	0.02
Plasma IL-6, Coef (95%CI)	0.46 (0.02–0.90)	0.04
Score, per 1- unit increment		
Unadjusted OR (95%CI)	3.24 (2.22–4.73)	<0.01
Adjusted† OR (95%CI)	2.72 (1.86–3.99)	<0.01
c Statistics		
Best-fit clinical model, AUC (95%CI)	0.731 (0.668–0.794)	0.02
Best-fit clinical model + multimarker score, AUC (95%CI)	0.808 (0.755–0.861)	
IDI (95%CI)	0.125 (0.084–0.167)	<0.01
Continuous NRI (95%CI)	0.672 (0.437–0.907)	<0.01
Categorical NRI (95%CI)	0.249 (0.122–0.375)	<0.01

*IDI* integrated discrimination improvement, *NRI* net reclassification improvement

*Levels of cytokines were standardized to the same scale (mean = 0, SD = 1)

†Analysis adjusted for age and stroke severity on admission

**Fig. 2:**
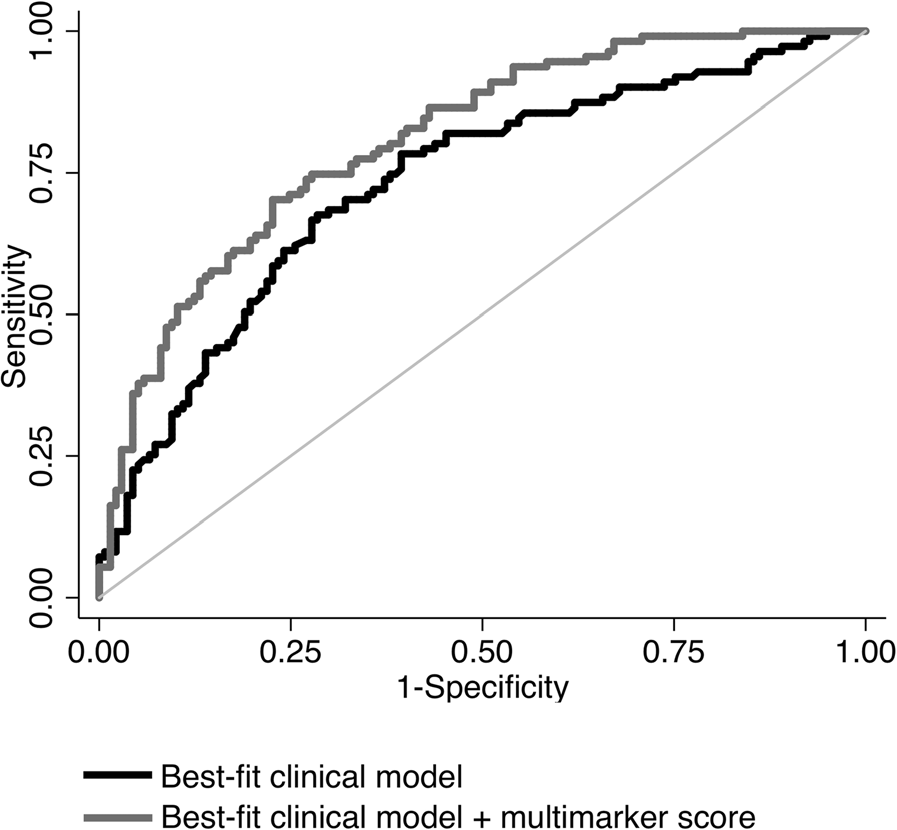
Receiver operating characteristics. Black line represents the performance of the best-fit clinical model to discriminate patients with poor outcome. Gray line shows the performance of the enhanced model after addition of multimarker score

In the multivariate analysis adjusted for age and stroke severity, patients with higher multimarker score had 2.72-times per unit increased risk of worse outcome (95%CI 1.86–3.99, *p* < 0.01). After addition of multimarker score to the best-fit clinical model, we observed improvement in discrimination of poor outcome (Fig. 2), supported by the increase in AUC (*p* = 0.02) and IDI (*p* < 0.01) (Table 3). The model with the multimarker score was well-calibrated (Hosmer-Lemeshow χ2 *p* = 0.98). Multimarker score also performed better than each of cytokines added to the best-fit clinical model (*p* < 0.05): IL-12p70 (AUC = 0.768), IL-10 (AUC = 0.750), IL-8 (AUC = 0.746), TNFα (AUC = 0.750), IP-10 (AUC = 0.750), IL-1β (AUC = 0.741), and plasma IL-6 (AUC = 0.752).

In addition, multimarker score significantly improved the classification accuracy for poor outcome compared to the best-fit clinical model as evidenced by categorical NRI (0.249, *p* < 0.01) and continuous NRI (0.672, *p* < 0.01). Reclassification tables are shown in Additional file 3: Table S2. Among patients with poor outcome, 18.0% (*n* = 20) were correctly upclassified versus 6.3% (*n* = 7) incorrectly downclassified. In the good outcome group 21.9% (*n* = 30) were correctly downclassified whereas 8.8% (*n* = 12) were incorrectly upclassified by multimarker score.

## Discussion

Our study showed that ex vivo cytokine synthesis after whole blood stimulation with LPS differs between stroke patients with good outcome and patients with poor outcome. We found that the decreased release of TNFα, IL-1β, IP-10, and IL-12 and the increased release of IL-10 and IL-8 are associated with poor functional outcome.

Emsley et al. studied ex vivo cytokine synthesis in 36 acute ischemic stroke patients [8]. They found the decreased TNFα, IL-1β, and IL-6 production after in vitro blood stimulation in non-survivors and patients with poor functional outcome. Recently, we reported that the reduced release of TNFα and IP-10 is independently from age and stroke severity associated with worse prognosis after stroke [9].

The current study provides a new piece of information about a relationship between ex vivo synthesized cytokines and stroke outcome. It demonstrates the decreased production of IL-12 and increased release of IL-10 and IL-8 in patients with unfavorable prognosis. Interleukin-12 is a pro-inflammatory cytokine that plays a key role in resistance to infections by modulating the activity of natural killer cells and T cells [25]. Interleukin-10 is a prototypic anti-inflammatory cytokine that limits the immune response to pathogens [26]. In stroke patients, higher plasma level of IL-10 is associated with the increased risk of infections [27] and lack of response to preventive antibacterial therapy [28]. Thus, the decreased IL-12 and increased IL-10 release from blood cells might contribute to post-stroke immunodepression.

Interleukin-8 is a chemokine involved in the recruitment and activation of neutrophils [29]. Neutrophils play an important role in ischemic brain injury [30]. Excessive neutrophil activation by IL-8 could contribute to greater brain damage after stroke and consequently leads to worse outcome.

Consistently with previous reports, we also observed the elevated level of circulating IL-6 in patients with poor outcome [6, 7].

We did not find any association between ex vivo synthesized IL-6 and functional outcome. Emsley et al. reported the correlation between the reduced IL-6 production and worse outcome after stroke [8]. This discrepancy between studies could be explained by several methodological differences including the sample size, the method of blood stimulation, and statistical approach. First, Emsley et al. measured cytokine production at several timepoints in the first week after stroke. Only minimal cytokine production was used to assess a correlation between cytokine synthesis and outcome. Second, Emsley et al. used much higher dose of LPS to stimulate blood cells (200 ng/mL vs 10 ng/mL). Third, the sample size differs between these two studies (36 vs 248 patients). Finally, we dichotomized outcome score and performed uni- and multivariate logistic regression analysis, whereas Emsley et al. correlated cytokine level with the mRS score.

Cluster analysis revealed interactions between synthesized cytokines, plasma IL-6, and stroke outcome. We identified three groups of patients with distinct cytokine profiles. The group with the worst outcome demonstrated relatively high synthesis of IL-10, IL-8, IL-1β, and IL-6 and low synthesis of IL-12, IP-10, and TNFα accompanied by high circulating IL-6 level. The group with the best prognosis showed relatively high synthesis of TNFα, IP-10, IL-12, IL-1β, and IL-6 together with low synthesis of IL-10 and IL-8. Plasma level of IL-6 was relatively low in this group. Patients with intermediate outcome had relatively low synthesis of all cytokines accompanied by low circulating IL-6. Our findings suggest that stroke outcome may depend on the interplay between stroke-induced immunodepression and systemic inflammation. The relatively low production of pro-inflammatory cytokines was associated with comparatively good outcome if immunodepression was not accompanied by systemic inflammation (low circulating IL-6). In experimental models of stroke, systemic inflammation is linked to poor outcome via permanent disruption of the blood-brain barrier, neutrophil mobilization and infiltration into the ischemic brain, impaired reperfusion, and enhanced platelet activity [10]. Immunodepression may worsen outcome by increasing the incidence of systemic infections [1]. Our cluster analysis also underlines the association between ex vivo IL-10 and IL-8 production and outcome. In contrast to patients with the best and intermediate prognosis, patients with the worst outcome had high synthesis of these cytokines.

Although observational studies are not able to demonstrate a causative relationship between cytokine synthesis and stroke outcome, both systemic inflammation and immunodepression could be considered as a potential therapeutic target. Agents that target components of IL-6 signaling pathway (e.g., monoclonal antibodies against IL-6 or IL-6 receptor or Janus kinases inhibitors) might attenuate systemic inflammation [31]. In healthy men volunteers who received endotoxin intravenously, immunodepression was partially reversed by subcutaneous administration of interferon-ɣ [32]. This treatment increased ex vivo LPS-induced TNFα release. In vitro β-glucan, a component of Candida albicans, partially reversed the LPS-induced immunodepression [33]. Ex vivo β-glucan treatment of monocytes from volunteers receiving LPS injection re-instanced their capacity for cytokine synthesis [31]. Of note, it remains unclear if reversion of post-stroke immunodepression will result in better outcome.

The accurate prediction of outcome after stroke is important in clinical practice. Biomarkers may facilitate stroke prognosis. Whiteley and colleagues proposed desirable features of a biomarker for stroke prognosis: (1) independent association of with outcome, (2) statistically significant predictive value added to a clinical model, and (3) improvement of the patients’ classification [22].

Blood-derived inflammatory biomarkers might provide useful information to improve prediction of outcome after stroke [34]. In the second part of our study, we assessed the prognostic value of cytokine-based multimarker score. Our multimarker score included ex vivo synthesized IL-12, IL-10, TNFα, and plasma IL-6. This multimarker score was independently associated with outcome after adjusting for the two most important stroke prognosticators: age and stroke severity. The addition of this score to the clinical model improved discrimination and reclassification. The multimarker score provided greater improvement in outcome prediction than individual cytokines. Although direct comparison between studies is difficult due to different methodologies, our multimarker score combining synthetized cytokines and plasma IL-6 seems to have better predictive value than other inflammatory markers including circulating IL-6, C-reactive protein, or fibrinogen [6, 7, 22, 35]. Given our results, it would be interesting to investigate in future studies the predictive ability of combination of ex vivo synthesized cytokines with other circulating inflammatory markers such as C-reactive protein or fibrinogen.

We need to address several limitations of our study. First, our results need confirmation in an independent population. Second, we performed whole blood stimulation 3 days after stroke. The prognostic value of our biomarkers measured early after stroke onset needs to be determined. Third, there are no widely acceptable thresholds to calculate category-based NRI. Similar to previous studies [7, 35], we applied stringent thresholds which are relevant for stroke clinical practice. Less rigorous thresholds could provide different results. Therefore, an optimal value of thresholds should be further tested in future studies.

We are aware that our novel biomarker is not ready for use in daily clinical practice. The necessity of blood culture in appropriate laboratory conditions strongly limits the technical feasibility of our biomarker. However, commercially available tubes with cell culture media and immune stimulant may offer a practical solution to monitoring cytokine synthesis without specialized technical experience [36]. They allow performing blood stimulation in room air using a heating block. Minimal sample handling and low amount of blood needed for stimulation are other advantages which may facilitate wider use of whole blood stimulation in clinics. It would be interesting to assess in future studies the combination of the cytokine-based composite biomarker with other non-inflammatory biomarkers (e.g., copeptin).

## Conclusions

In conclusion, the decreased release of some pro-inflammatory cytokines (IP-10, TNFα, IL-1β, and IL-12p70) and increased release of IL-10 and IL-8 after ex vivo blood stimulation with LPS are related to poor outcome after stroke. This association is modulated by plasma IL-6. Cytokine-based multimarker score composed of ex vivo released IL-12, IL-10, TNFα, and plasma IL-6 adds prognostic value to clinical model for predicting stroke outcome.

## Supplementary information

**Additional file 1: Figure S1**. NbClust’s optimal number of clusters. Among all indices provided by NbClust package in R, 12 indices proposed 3 as the best number of clusters

**Additional file 2: Table S1**. Comparison of baseline characteristics and levels of cytokines between clusters

**Additional file 3: Table S2**. Reclassification table for poor outcome

## Data Availability

The datasets used during the current study are available from the corresponding author on reasonable request.

## Abbreviations

AUC: Under receiver operator curves IDI Integrated discrimination improvement IL Interleukin IP-10 Interferon-gamma-inducible protein 10 IQs Interquartiles mRS Modified Rankin Scale NIHSS National Institute of Health Stroke Scale NRI Net reclassification improvement TNFα Tumor necrosis factor alpha
