# Supplementary figures and images for: The specific ex vivo released cytokine profile is associated with ischemic stroke outcome and improves its prediction
Source: J Neuroinflammation. 2020 Jan 6;17:7. doi: 10.1186/s12974-019-1691-1 (PMC6945431; doi:10.1186/s12974-019-1691-1)

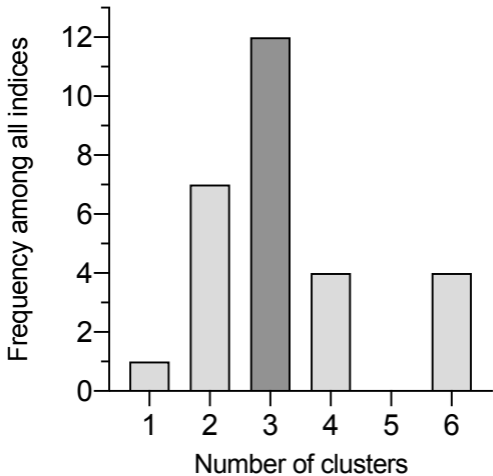

Supplement: Supplementary file 1 — Additional file 1: Figure S1. NbClust’s optimal number of clusters. Among all indices provided by NbClust package in R, 12 indices proposed 3 as the best number of clusters [file 12974_2019_1691_MOESM1_ESM.pdf]
