# Supplementary material for: The specific ex vivo released cytokine profile is associated with ischemic stroke outcome and improves its prediction
Source: J Neuroinflammation. 2020 Jan 6;17:7. doi: 10.1186/s12974-019-1691-1 (PMC6945431; doi:10.1186/s12974-019-1691-1)
Supplement: Supplementary file 2 — Additional file 2: Table S1. Comparison of baseline characteristics and levels of cytokines between clusters [file 12974_2019_1691_MOESM2_ESM.docx]

**Table S1.** Comparison of baseline characteristics and levels of cytokines between clusters.

|  | | **Cluster 1**  **n=68** | **Cluster 2**  **n=65** | **Cluster 3**  **n=115** | **p-value** |
| --- | --- | --- | --- | --- | --- |
| Age, median (IQs) | | 67 (60-74.5) | 73 (65-80) | 67 (60-80) | 0.04 |
| Female, n (%) | | 15 (22.1) | 28 (43.1) | 59 (51.3) | <0.01 |
| Hypertension, n (%) | | 57 (83.8) | 55 (84.6) | 82 (71.3) | 0.05 |
| Diabetes mellitus, n (%) | | 22 (32.4) | 19 (29.2) | 29 (25.2) | 0.57 |
| Atrial fibrillation, n (%) | | 19 (27.9) | 24 (36.9) | 28 (23.4) | 0.20 |
| Myocardial infarction, n (%) | | 10 (14.7) | 12 (18.5) | 12 (10.4) | 0.31 |
| Previous stroke, n (%) | | 7 (10.3) | 9 (13.9) | 14 (12.2) | 0.82 |
| Current smoking, n (%) | | 21 (30.9) | 14 (21.5) | 29 (25.2) | 0.46 |
| NIHSS score on admission, median (IQs) | | 7 (4-14) | 16 (6-19) | 9 (5-16) | <0.01 |
| Thrombolysis, n (%) | | 43 (63.2) | 31 (47.7) | 64 (55.7) | 0.20 |
| Thrombectomy, n (%) | | 18 (26.5) | 17 (26.2) | 30 (26.1) | 1.00 |
| Poor outcome, n (%) | | 17 (25.0) | 45 (69.2) | 49 (42.6) | <0.01 |
| Etiology | |  |  |  | 0.22 |
|  | Large vessel disease, n (%) | 12 (17.7) | 18 (27.7) | 36 (31.3) |  |
|  | Small vessel disease, n (%) | 3 (4.4) | 2 (3.1) | 8 (7.0) |  |
|  | Cardioembolic, n (%) | 21 (30.9) | 24 (36.9) | 28 (24.4) |  |
|  | Undetermined, n (%) | 31 (45.6) | 19 (29.2) | 38 (33.0) |  |
|  | Other, n (%) | 1 (1.5) | 2 (3.1) | 5 (4.4) |  |
| **Cytokines** | | | | | |
| Ex vivo stimulation | | | | | |
|  | IL-12p70, pg/ml, median (IQs) | 8.1 (4.6-15.0) | 3.9 (0.4-5.4) | 3.2 (0-6.6) | <0.01 |
|  | IL-10, pg/ml, median (IQs) | 45.3 (32.7-65.8) | 95.8 (70.4-125.7) | 40.5 (28.0-60.6) | <0.01 |
|  | IL-6, pg/ml, median (IQs) | 14979  (11460-19238) | 17214  (13571-21973) | 8310  (6349-10941) | <0.01 |
|  | IL-1β, pg/ml, median (IQs) | 2057 (1466-2883) | 1940 (1280-2561) | 1120 (774-1484) | <0.01 |
|  | IL-8, pg/ml, median (IQs) | 1427(939-2069) | 3459 (2653-5141) | 1406 (944-2038) | <0.01 |
|  | TNFα, pg/ml, median (IQs) | 3535 (2701-4420) | 2665 (2066-3562) | 1782 (1232-2314) | <0.01 |
|  | IP-10, pg/ml, median (IQs) | 814 (592-1070) | 277 (149-477) | 277 (151-436) | <0.01 |
| Plasma | | | | | |
|  | IL-6, pg/ml, median (IQs) | 3.7 (2.0-7.0) | 18.3 (5.6-32.2) | 3.9 (1.9-7.6) | <0.01 |

IQs – interquartiles; NIHSS – National Institutes of Health Stroke Scale.
