# Supplementary material for: The specific ex vivo released cytokine profile is associated with ischemic stroke outcome and improves its prediction
Source: J Neuroinflammation. 2020 Jan 6;17:7. doi: 10.1186/s12974-019-1691-1 (PMC6945431; doi:10.1186/s12974-019-1691-1)
Supplement: Supplementary file 3 — Additional file 3: Table S2. Reclassification table for poor outcome [file 12974_2019_1691_MOESM3_ESM.docx]

**Table S2**. Reclassification table for poor outcome.

**Patients with poor outcome 3 months after stroke**

|  | Best-fit clinical model + multimarker score | | | | |
| --- | --- | --- | --- | --- | --- |
| Best-fit clinical model | 0%-10% | 10%-50% | 50%-90% | 90%-100% | % Reclassified |
| 0%-10% | 0 | 0 | 0 | 0 | 0 |
| 10%-50% | 0 | 32 | 11 | 2 | 29 |
| 50%-90% | 0 | 7 | 52 | 7 | 21 |
| 90%-100% | 0 | 0 | 0 | 0 | 0 |

**Patients with good outcome 3 months after stroke**

|  | Best-fit clinical model + multimarker score | | | | | |
| --- | --- | --- | --- | --- | --- | --- |
| Best-fit clinical model | 0%-10% | 10%-50% | 50%-90% | | 90%-100% | % Reclassified |
| 0%-10% | 3 | 0 | 0 | 0 | | 0 |
| 10%-50% | 17 | 74 | 10 | 0 | | 27 |
| 50%-90% | 2 | 11 | 18 | 2 | | 45 |
| 90%-100% | 0 | 0 | 0 | 0 | | 0 |
